# Exploring the costs of phenotypic plasticity for evolvable digital organisms

**DOI:** 10.1038/s41598-023-50683-3

**Published:** 2024-01-02

**Authors:** Karine Miras

**Affiliations:** Department of Computer Science, Vrije Universiteit Amsterdam, Amsterdam, The Netherlands

**Keywords:** Computational science, Computer science, Evolutionary developmental biology, Experimental evolution

## Abstract

Phenotypic plasticity is usually defined as a property of individual genotypes to produce different phenotypes when exposed to different environmental conditions. While the benefits of plasticity for adaptation are well established, the costs associated with plasticity remain somewhat obscure. Understanding both why and how these costs arise could help us explain and predict the behavior of living creatures as well as allow the design of more adaptable robotic systems. One of the challenges of conducting such investigations concerns the difficulty of isolating the effects of different types of costs and the lack of control over environmental conditions. The present study addresses these challenges by using virtual worlds (software) to investigate the environmentally regulated phenotypic plasticity of digital organisms. The experimental setup guarantees that potential genetic costs of plasticity are isolated from other plasticity-related costs. Multiple populations of organisms endowed with and without phenotypic plasticity in either the body or the brain are evolved in simulation, and organisms must cope with different environmental conditions. The traits and fitness of the emergent organisms are compared, demonstrating cases in which plasticity is beneficial and cases in which it is neutral. The hypothesis put forward here is that the potential benefits of plasticity might be undermined by the genetic costs related to plasticity itself. The results suggest that this hypothesis is true, while further research is needed to guarantee that the observed effects unequivocally derive from genetic costs and not from some other (unforeseen) mechanism related to plasticity.

## Introduction

In nature, the traits of living creatures are not coded independently in their genetic material. One obvious reason for this is compression: the reuse of genetic material helps to simplify this ‘optimization problem’ by solving small parts of the problem and then combining solutions accordingly. For instance, while only approximately 30, 000 genes code all traits of the human phenotype^1^, the human brain alone consists of trillions of neurons^2^. While compression is expedient for simple cases of reuse, e.g., coding for multiple fingers, it also helps to produce greater benefits: the remarkable levels of intelligence observed in nature are not simply a result of the discovery of how to produce certain traits but rather, most importantly, derive from determining when to express these traits^3^.

The environment surrounding organisms is constantly changing, and therefore, organisms can encounter countless environmental conditions over their lifetime. These changes can vary from internal (intra-phenotype) to external changes, such as weather, terrain, predators, and interspecies interactions. To cope with these changes, different dimensions of phenotypic development are observed in nature.

Phenotypic plasticity “is usually defined as a property of individual genotypes to produce different phenotypes when exposed to different environmental conditions”^4^. The term encompasses morphological, physiological, and behavioral changes and has been utilized in a very broad sense throughout extant literature^5^, including even the fields of learning^6^ and body training^7^. The more specific usage of the term refers to the environmentally regulated expression of different phenotypes from the same genotype. One example of this is polyphenism^8^: irreversible phenotypic differentiation, e.g., castes in social insects and sex determination in reptiles. Another example is acclimatization^9^: reversible phenotypic changes triggered by environmental cues, e.g., Passerine birds that change their musculature to cope with the winter.

Phenotypic plasticity can be highly adaptive, provided that the plastic response is sufficiently fast, accurate, and not too costly^10^. It can increase fitness by allowing organisms to adjust their traits in response to environmental changes and might facilitate evolution, increasing diversity and speciation^11^. It has been argued that plasticity is helpful in colonizing new environments and dealing with geographical shifts^12^. Furthermore, researchers have demonstrated that plasticity can be an adaptive response to climate change, significantly diminishing the risk of extinction^13^.

While the benefits of plasticity for adaptation are well established, the constraints surrounding plasticity remain somewhat obscure^14^. Such constraints are referred to as *costs* and *limits*. Although these costs and limits have been divided into distinct categories in the literature, their exact definitions diverge, and their mechanisms are not yet well understood^14–16^. One definition describes them as follows: “Costs of plasticity are fitness deficits associated with the plastic genotypes relative to fixed genotypes producing the same mean phenotype in a focal environment, and limits of plasticity are functional constraints that reduce the benefit of plasticity compared to perfect plasticity”^14^. This means that in the case of limits, a plastic genotype can not express the (extreme) traits like a non-plastic genotype does, while in the case of costs, the traits can be obtained, but the fitness is reduced for other reasons. Another definition argues that expressing suboptimal (‘wrong’) phenotypes in a given environment could be viewed as a cost, and that it is not straightforward to separate costs from limits^17^.

Although plasticity-related costs have been studied for years, the evidence for costs is variable^18–20^, and the literature has produced a mixed picture, with a few studies reporting evidence for costs while others observe no costs^16,18,19,21,22^. Furthermore, even when there is evidence for costs, it remains unclear what caused them, as a mechanistic understanding of their nature is lacking^23^. The confusion in the literature ranges from the difficulty of detecting certain costs, e.g., the costs for flowering time^24^, to the observation of what seems to be the opposite of limits of plasticity, i.e., plastic responses resulting in traits that are more extreme than those of the focal environment^25^.

There is a wide range of challenges that make it difficult to study plasticity-related costs, which explains some of the disagreement among the studies. One of these challenges pertains to the potential differences between the environments where the acclimatization response evolved and the environments used for tests and analysis, while another difficulty concerns the lack of control of the types of plasticity^26^. Moreover, there are multiple types of costs, which could hardly be experimentally isolated in natural life systems. Five classical costs have been defined in the literature: *production costs*, *maintenance costs*, *information-acquisition costs*, *developmental-instability costs*, and *genetic costs*^14,17^. The present study addresses these challenges through Artificial life (ALife). ALife has been characterized as the study of “life as it could be”^27^, and it involves the design and investigation of artificial living systems in different levels of organization (molecular, cellular, organismal, populational) and mediums (hardware, software, wetware)^28^. Advances in ALife are expedient for designing artificially intelligent systems as well as for understanding naturally intelligent systems.

Using digital organisms in ALife studies to investigate biological phenomena^29^ is in line with the generic approach of employing computational models to expand biological knowledge, e.g., the application of abstract models of Gene Regulatory Networks for simulating and understanding gene interactions^30^. Models of evolution have been successfully used to research the role of neural modularity^31^, the emergence of adaptive behavior^32^, communication^33^, etc. The essence of this approach is to construct models that implement fundamental biological principles in new substrates—software or hardware—instead of ‘wetware’. The specific mechanisms for implementing such principles (e.g., selection, reproduction, gene regulation) can be substrate-specific without the limitation of having to use mechanisms identical to biological ones. This approach is relevant for biology because these models assimilate general biological principles, although there can be differences between natural and artificial evolutionary systems^34^.

Specifically, there are at least three benefits of using ALife to investigate natural phenomena. First, the customization of multiple properties is possible within an artificial life system. Clearly, this does not guarantee the emergence of specific phenomena but rather makes it possible to deliberately include or exclude certain mechanisms that may (or may not) lead to the emergence of these phenomena. This flexibility is relevant for studying phenotypic plasticity costs because it makes it possible to isolate effects within different types of costs. Second, it enables control over environmental conditions. Finally, it allows for cost-effective repetition of experiments to achieve statistical significance.

Artificial organisms that possess both a body and a brain have been evolved in several studies since 1994^35–37^. However, scarce attention has been given to the environmental regulation of phenotypic plasticity. Relatively few examples of studies related to development and phenotypic plasticity are found in the literature^38–46^, and none of these examine the costs of plasticity. The current experimental setup evolves multiple populations of organisms endowed with and without phenotypic plasticity in their bodies (morphologies) and brains (controllers) in simulation. The resulting experiments are used to investigate the *genetic costs* of phenotypic plasticity (specifically acclimatization) for evolvable digital organisms. Genetic costs are “costs that result directly from linkage between loci affecting plasticity and loci with negative fitness effects, (negative) pleiotropic effects of loci affecting plasticity and other traits, or (negative) epistatic interactions among loci affecting plasticity and other loci^17^”. Genetic linkage^47^ refers to the tendency of genetic sequences located close together on a chromosome to be inherited together during sexual reproduction. *Pleiotropy*^48^ concerns genes that are able to influence more than one trait in the phenotype. *Epistasis*^49^ refers to the interaction between genes, namely the fact that the effect of a gene may depend on either the presence or absence of another gene or genes. Both pleiotropy and epistasis are possible within the utilized genetic representation. Linkage does not apply because reproduction within the current system is asexual.

Here, a hypothesis is posited that despite the potential of phenotypic plasticity, its benefits may be undermined by plasticity-related genetic costs. More specifically, we expect that for both body and brain, acclimatization will take place at the cost of fitness deficits.

## Experimental setup

**Figure 1:**
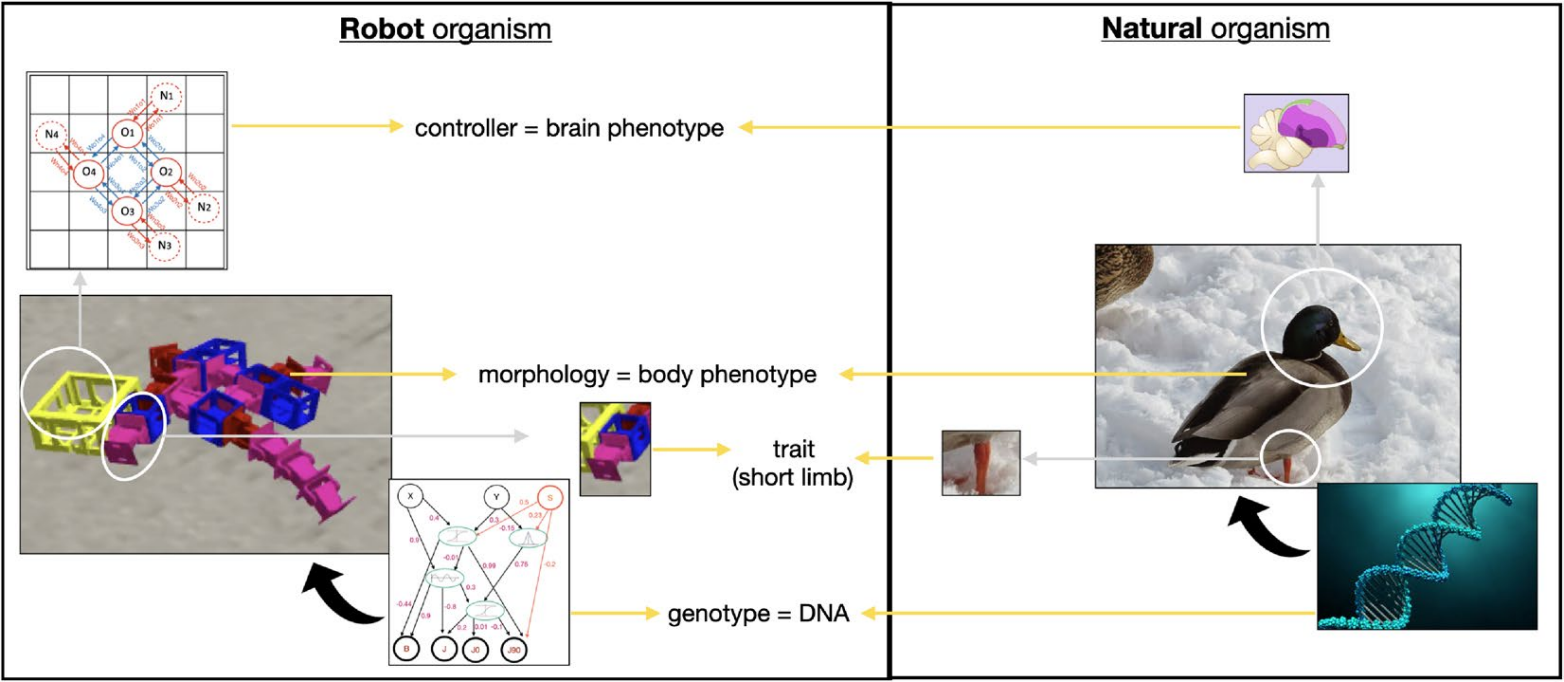
Link between the ALife abstractions utilized and their original concepts in biology. More details about each concept are provided in the remaining of this section and the “Methods” section.

Given that this work is aimed at audiences from various backgrounds and, as such, there can sometimes be a terminology mismatch, the key terms used throughout the text are clarified in Supplementary Information (Glossary). Additionally, Fig. 1 illustrates the main links between the ALife abstractions utilized and their original concepts in biology.

The studied organisms are modular robots. Their bodies are composed of a group of assembled modules, and their brains are networks that control the motor modules in their bodies. Phenotypic plasticity is applied by changing the body modules or the structure and weights of the networks. More details are provided in the “Methods” section.

All experiments (under each setup) described here were repeated 20 times independently for statistical significance. The experiments were carried out using six different setups that combined three aspects: *phenotypic plasticity* (with or without), *phenotypic component* (body or brain), and *desired behavior* (locomotion left, right, or down). In these experiments, the organisms had to perform a pair of behaviors throughout the 60 s of their lifetime: in a plane terrain environment, they were supposed to locomote in direction A in the first 30 s of their life and then locomote in direction B in the last 30 s of their life. To ensure the comparability of the behavioral metrics, the organisms were reset to their starting position before moving on in direction B. In this way, for both directions A and B, the initial conditions of the body morphology were the same.

The pairs of directions were *right-left* and *right-down*. Methods with and without phenotypic plasticity were applied for both pairs, while plasticity could be found either in the body and the brain or only in the brain. Additionally, a *baseline* experiment was included in which the organisms had to perform a single behavior for 30 s: locomoting right. The baseline represents a case in which the organisms evolve in what is called a *focal environment* in biology, as opposed to having to cope with multiple environmental conditions. Evidently, there was no plasticity in the focal environment.

**Figure 2:**
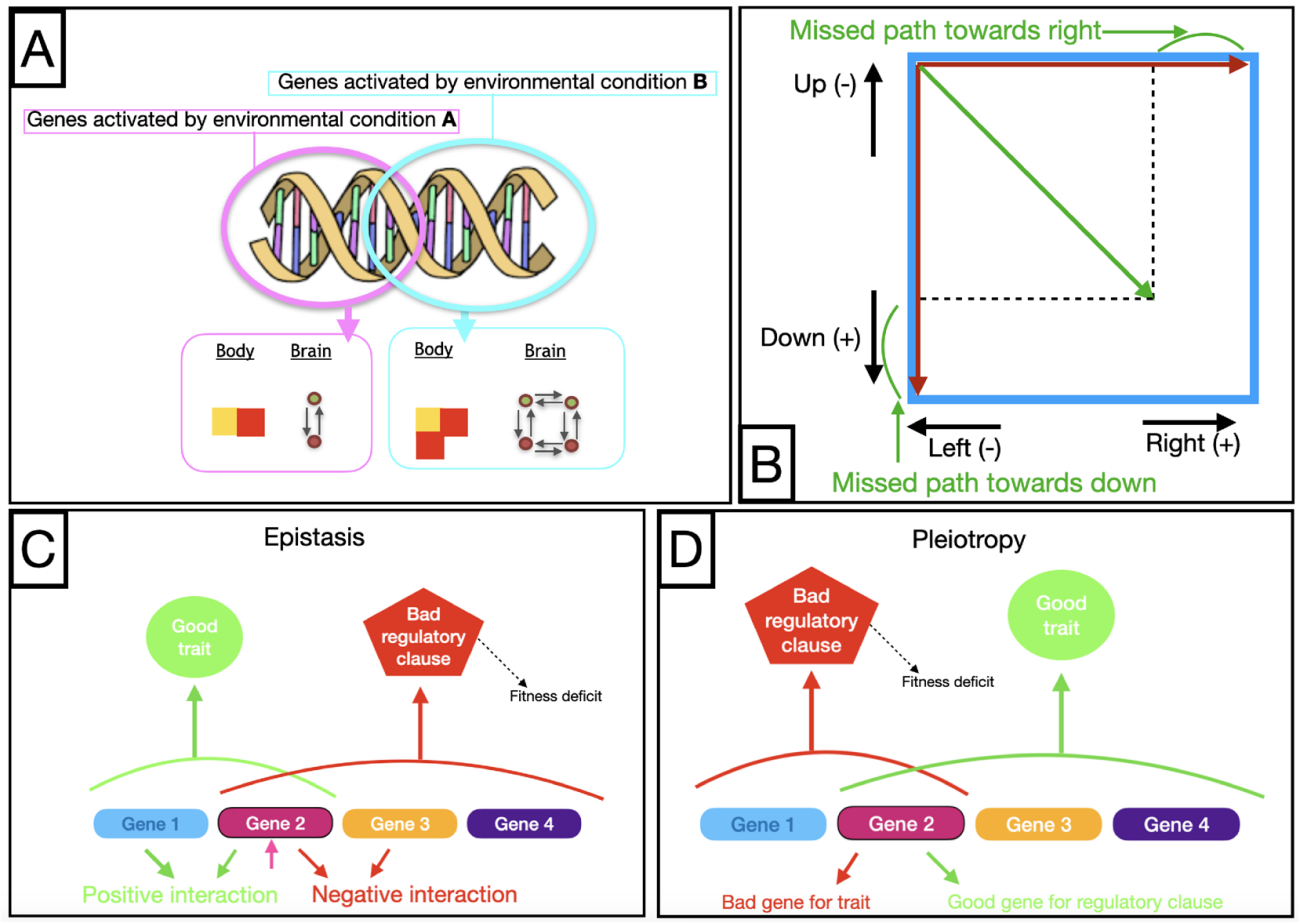
(**A**) Didactic illustration of the environmentally regulated phenotypic plasticity. (**B**) In the *right-down* pair, a perfect behavioural repertoire would consist of moving in a straight line towards the right when the expected behaviour is going right and moving in a straight line towards down when the expected behaviour is going down. The lines resulting from these two trajectories form an angle of 90$^\circ$ (red lines). If a non-plastic organism were to attain a perfect balance between displacing right and down, this would require moving in a 45$^\circ$ diagonal between the two trajectories (green line)—this diagonal would miss more than a fourth of the path in each direction. (**C** and **D**) Didactic examples of genetic costs: negative effects of gene interactions (epistasis) or reuse (pleiotropy) can cause fitness deficits.

Phenotypic plasticity is regulated by the environment: when organisms sense states in the environment, these states are inputted to their genotypes, which in turn use these states to regulate the expression of genetic material as phenotypic traits (body and/or brain). Generally, such states might be composed of any internal or external environmental factors that could be sensed or perceived by the organism. In the current setup, this is provided as a signal from a ‘central system’, and the signal represents the direction in which the organism should go (desired behavior). More details are described in the “Methods” section. A didactic illustration of the environmentally regulated phenotypic plasticity is displayed in frame A of Fig. 2.

The design choices made when forming the pairs of desired directions in the experimental setup aimed at composing extreme cases: a case in which plasticity is paramount and a case in which its benefits might not compensate for the costs involved. The objective was to clearly demonstrate both the potential and limitations of the studied plasticity approaches: the first case allows us to validate that the regulatory system is functional, while the second challenges its capacity. Plasticity would be evidently advantageous to the *right-left* pair because this case requires mutually exclusive behaviors, meaning that behavior that succeeds at locomoting in one direction fails inversely proportionally at locomoting in the other: by displacing itself maximally to the right, an organism succeeds maximally at displacing to the right but fails maximally at displacing to the left, and vice versa. This pair illustrates environmental conditions in which behavioral success is either impossible or highly unlikely because the nature of the required behaviors (or phenotypic traits) is too divergent. As for the *right-down* pair, it involves directions that can partially gain from each other: a behavior that follows a diagonal line between right and down succeeds (at some level) in displacement both towards the right and towards down. On the other hand, this strategy incurs a ‘loss’ resulting from imperfect trajectories (Fig. 2B). Importantly, it is not clear if this loss would be sufficient to create pressure toward phenotypic plasticity. For instance, the potential gain of approaching the full trajectory might not pay off when contrasted with the potential genetic costs.

The idea of organisms carrying out desired behaviors regulated by signals, e.g., going in direction A when receiving signal 1, is a simplification to enable a simple and clear experimental setup. To link this setup to a more natural context, the following analogies are proposed. The pairs of directions could represent vital locations: the organism might be in a region (A) that is not so rich in food; from region A, if the organism has gathered food, it should go to its nest that is in the east; if the organism has not gathered food, however, it should go to a region more abundant in food in the west. Moreover, the paths toward the east and west could be inhabited by different species of predators. Furthermore, let us assume that the species in the east are better at identifying darker colors, and those in the west are better at lighter colors. Concerning body plasticity, an organism that converges to an ‘in-between-solution’ (like the diagonal trajectory) would never become maximally invisible to any predators. Similarly, regarding brain plasticity, the eastern and western predators could have different abilities in detecting gait-related auditory cues, i.e., sensitivity to certain sound waves. Therefore, different neural structures could be needed to control the body while minimizing the production of detectable sound waves.

The success criterion (fitness) for assessing behavior measures the speed (cm/s) in the desired direction. Note that the term fitness here refers to a measure of quality calculated a priori^34^, as opposed to a posteriori, as in biology. It is used to select organisms for reproduction and survival. The final fitness function that consolidates performance between the two directions counts the number of Pareto-dominated solutions using the two fitness values: speed in direction A and speed in direction B.

Finally, the only plasticity costs allowed in the system are genetic costs (Fig. 2C,D), which can arise through pleiotropy and epistasis within the utilized representation (described in the “Methods” section). Other costs found in nature are maintenance, energetic costs of regulatory systems; production, costs of expressing a trait; information acquisition, costs incurred when obtaining information about environmental conditions; and developmental instability, costs associated with imperfect phenotype-environment matching^17^. In the current setup, however, there are no production costs because organisms are given infinite ‘energy,’ in the sense that they do not need to succeed in any behaviors, e.g., eating, to produce phenotypic changes. Additionally, there are no maintenance, information-acquisition, or developmental-instability costs because environmental signals are unconditionally provided from a central system to organisms, and their regulatory systems also function unconditionally and unequivocally.

## Results

An analysis is presented comparing the ability of each method to produce organisms capable of coping with environmental changes over the course of their lifetime. The results of these methods are compared to the baseline to determine whether the methods are able to produce similar traits and fitness to those they would produce if only one behavior were needed (focal environment). A video displaying some of the evolved organisms behaving in the environment is available at https://youtu.be/F6pcbR5RFpQ. The video summarizes this paper in a language accessible to the general public.

### Effects of plasticity on behavior

**Figure 3:**
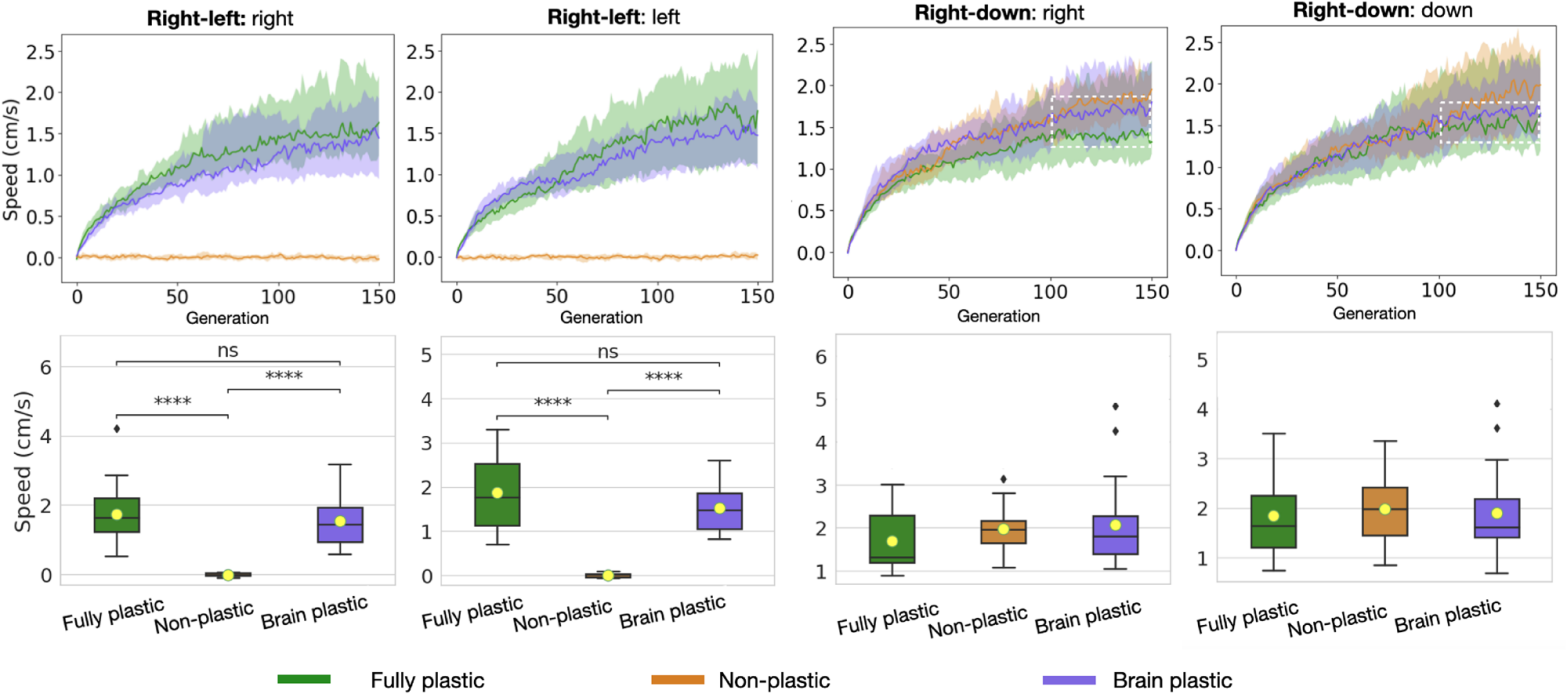
Fitness comparison. While the curves of the plastic methods grow until generation 100, for the next 50 generations, they oscillate without presenting any real growth and appear to have stagnated (highlighted by dotted white lines). *Speed* is averaged within the population for each generation using means and then averaged again amongst the experiment repetitions using quartiles (lines are the medians, and shades are Q25 and Q75). This same data aggregation applies to the other line charts in this paper. The boxes compare populations in the final generation using Wilcoxon tests with significance: ****$p <= 0.0001$. After Bonferroni corrections for three comparisons, the significance threshold is $p< 0.016$.

Plastic organisms produced a much better performance in both directions of the *right-left* pair than non-plastic organisms, demonstrating that the regulatory system is functional (Fig. 3). Considering the fitness function, the fact that the non-plastic organisms, on average, can barely locomote is not unexpected: the solutions are random in the beginning, insofar as there is an equal probability that the organisms displace slightly in the correct or incorrect direction, so that their displacement is close to zero on average; because the behaviors involved are mutually exclusive, the non-plastic organisms never dominate each other, and therefore the zero-average region is never abandoned. In contrast, no significant difference in performance was observed between plastic and non-plastic organisms in any direction for the *right-down* pair. Additionally, the speed in direction A was comparable to the speed in direction B for all the direction pairs and methods (Fig. 5). The ability of the non-plastic organisms to locomote under both conditions is linked to plasticity not being the only form of increasing *niche breadth*^50^, i.e., the spectrum of environmental conditions that a species can cope with.

**Figure 4:**
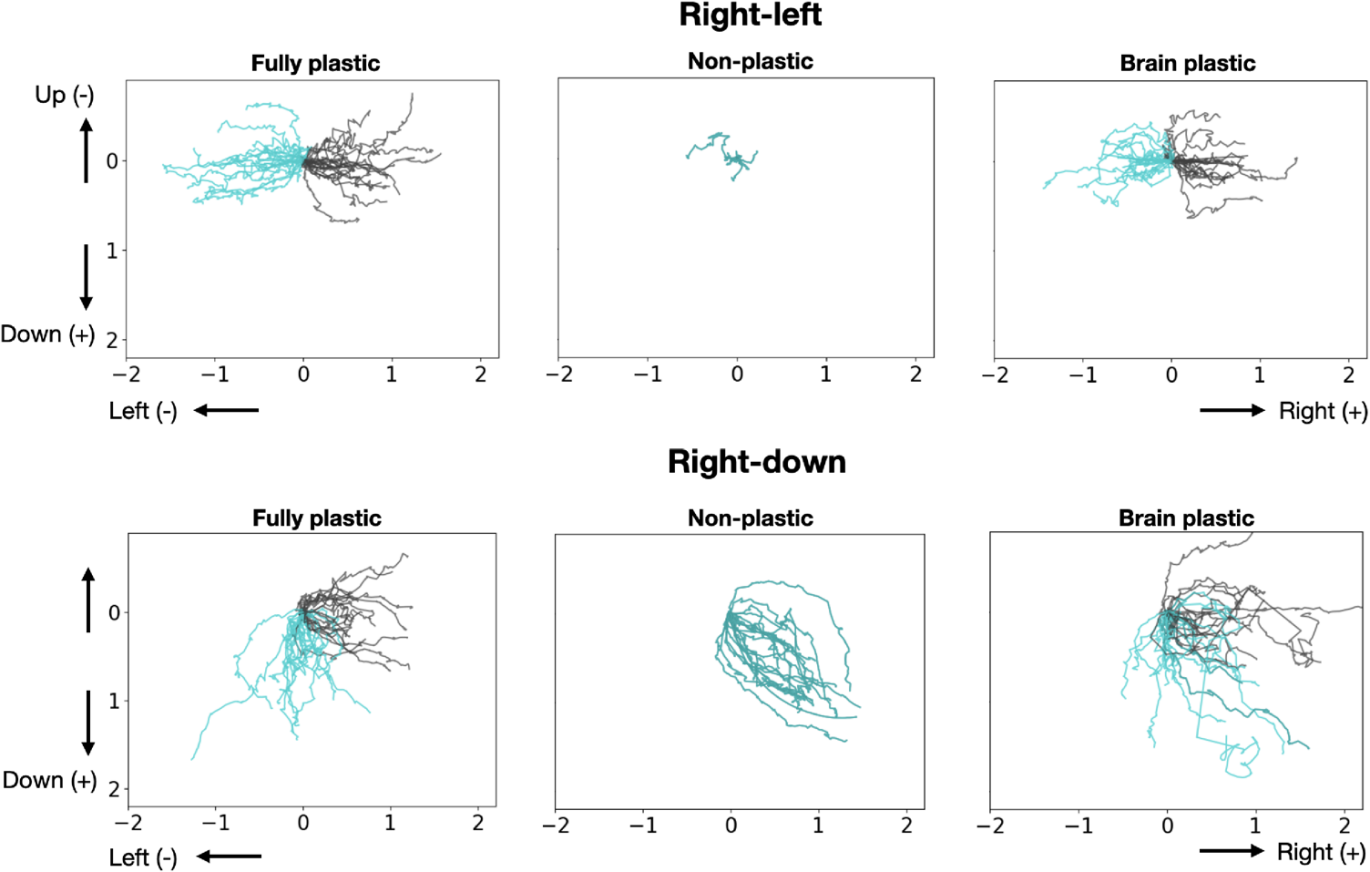
Trajectories of the best organism in the last generation of each experiment. The black and blue lines represent the trajectories of the first behaviour and second behavior of the directions pair, respectively. The trajectories of the experiments were drawn on the same picture, and the individual trajectories of *right-down* can be seen in Figs. 1, 2, and 3 of Supplementary Information. Interestingly, the brain plastic method produced identical trajectories towards both directions in two of the experiments with the *right-down* pair. This suggests that the regulated phenotypes are also identical or extremely similar, and therefore plasticity evolved to be idle.

These aforementioned behavioral differences are illustrated in Fig. 4, which shows the trajectories taken by the organisms when locomoting. As expected, the trajectories of the plastic organisms in the *right-left* pair follow opposite paths when they need to go either right or left, whereas the non-plastic organisms simply wobble in the middle. Furthermore, the trajectories of the plastic organisms in the *right-down* pair often spread toward the right or down more precisely, according to the goal direction, whereas the trajectories of the non-plastic organisms more frequently form a diagonal. Nevertheless, these diagonals appear to form longer paths than the trajectories of the plastic organisms. The visual inspection is aided by measuring the total displacement: non-plastic organisms are displaced more in terms of the sum of the *x* and *y* displacements (Supplementary Information Fig. 4). This displacement allows the points in space that are reached by these diagonals to be, on average, as distant from the initial points as the points reached by the plastic methods.

**Figure 5:**
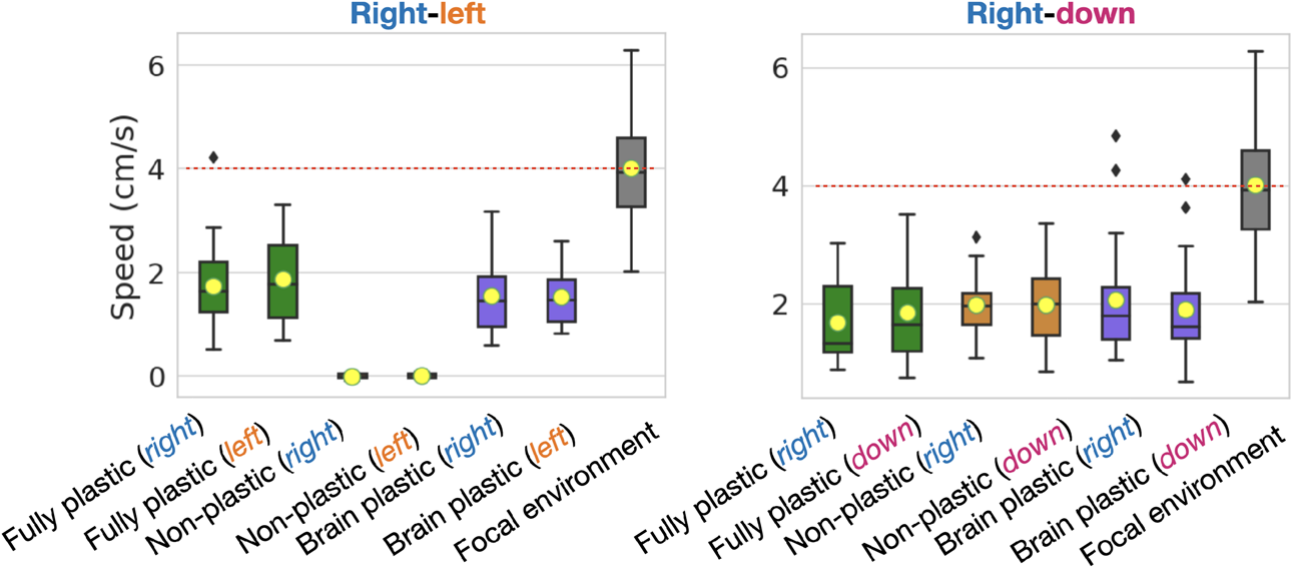
Fitness comparison between the focal environment (baseline) and the different setups in each condition.

Finally and most importantly, the speed of the organisms in the focal environment is much higher than the speed produced by any of the methods (Fig. 5). This loss in fitness is expected when comparing the focal environment to the non-plastic method because a single phenotype was ‘divided’ between pressures for different behaviors. Nevertheless, this loss was less expected from the plastic methods because they have the capacity to develop. However, as discussed in the next section, phenotypic plasticity seems to have incurred costs.

Comparing the focal environment with the other experiments means comparing populations that evolved in a single environment with populations that evolved in multiple environmental conditions instead. While one might consider this comparison “unfair”, the comparison is not about fairness but about understanding the constraints of plasticity: “Jack of all trades, master of none”. It is intuitive and sensible to consider that if an organism needs to solve multiple problems (express multiple traits plastically) instead of one, its performance might suffer degradation in one case or another (costs). Nevertheless, there are various ways in which this degradation could occur (different costs), and not so much is known about how they occur.

### Fitness landscape analysis

The previous analysis demonstrates that in addition to being unable to attain the fitness of the organisms in the focal environment, in addition to this, plastic organisms cannot always achieve a superior behavioral repertoire to non-plastic organisms. To investigate this, the data were inspected further by analyzing the fitness landscape via the following procedure. Novelty Search^51,52^ was carried out to explore the space of the possible organisms ‘agnostically’. The objective of the search was to find solutions as (behaviorally) different from each other as possible; the organisms resulting from the search were then measured in regard to body traits^53^; these traits were reduced to two principal components, and the average speed (between the two behaviors) was laid on the landscape of these components. Further details on this procedure can be found in the Supplementary Information (Procedure for landscape analysis).

**Figure 6:**
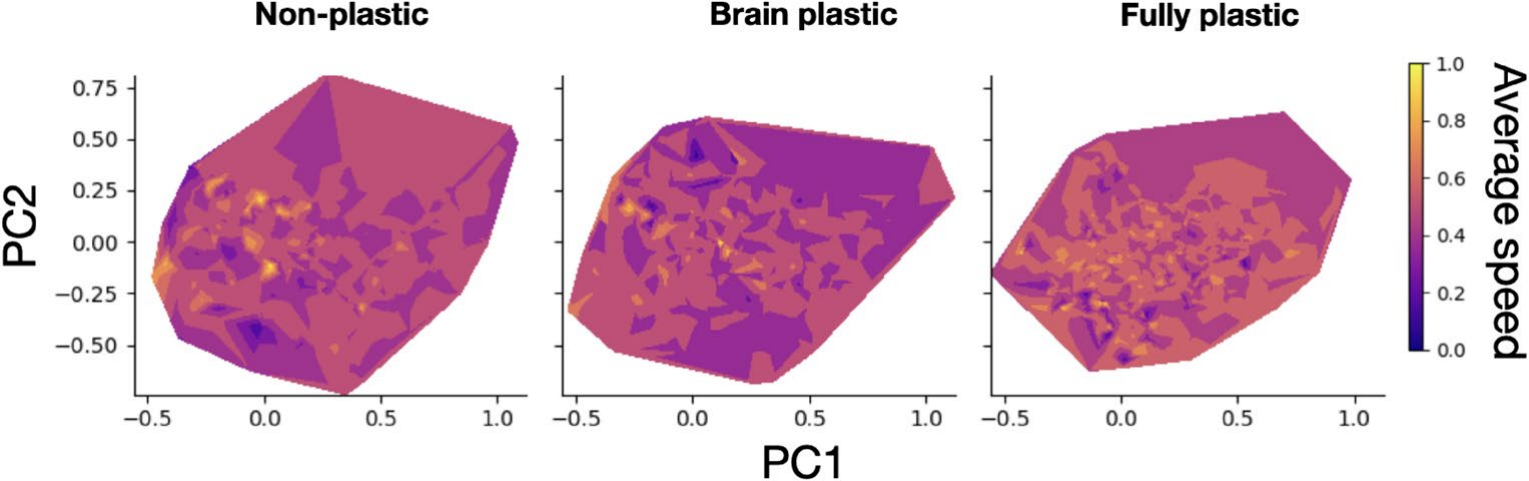
Fitness landscape comparison between methods for the ***right-down*** *pair.* Axes represent the components that encode body traits. The components were obtained by Principal Component Analysis of a set containing the data from all three methods. Average speed is the mean of speed in direction A and speed in direction B. The white area refers to values that did not occur.

The procedure produced the landscape illustrations presented in Fig. 6. Comparing the landscapes shows that for the *right-down* pair, the *fully plastic* method is more rugged, i.e., has more peaks (areas of high fitness) that are more spread out than the other methods have. This is aligned with ideas discussed in the literature about how pleiotropy and epistasis can result in ruggedness intensification^54,55^. These observed landscapes provide partial evidence that plasticity makes landscapes more rugged and, as such, more difficult to search. It is necessary to acknowledge here, however, that categorically concluding that the fitness landscapes of the plastic methods are more rugged would be an overstatement because it is not known if this is the case for all the possible dimensions, i.e., body and (especially) brain traits that were never formulated and therefore were not measured. Furthermore, the aggregation method applied makes it difficult to interpret the data; for example, speed was averaged using both behaviors as well as the traits in both conditions for the case of body plasticity. Notwithstanding, this examination reveals that, at least from these specific dimensions, there is a discernible difference between the studied landscapes. It is thus possible that plasticity influences the ‘complexity’ of the landscape and that this could have played a role in the shortcomings of plasticity. This evidence favors the conjecture put forward in the previous section that the potential gain of approaching the full trajectory might not pay off when contrasted with genetic costs. This can be argued through the following reasoning: genetic costs are characterized by negative pleiotropic and epistatic effects; pleiotropy and epistasis intensify ruggedness; the landscape of the fully plastic method appeared to be more rugged while the method did not fulfill the maximum theoretical plasticity potential (focal environment fitness).

### Body vs. brain plasticity

**Figure 7:**
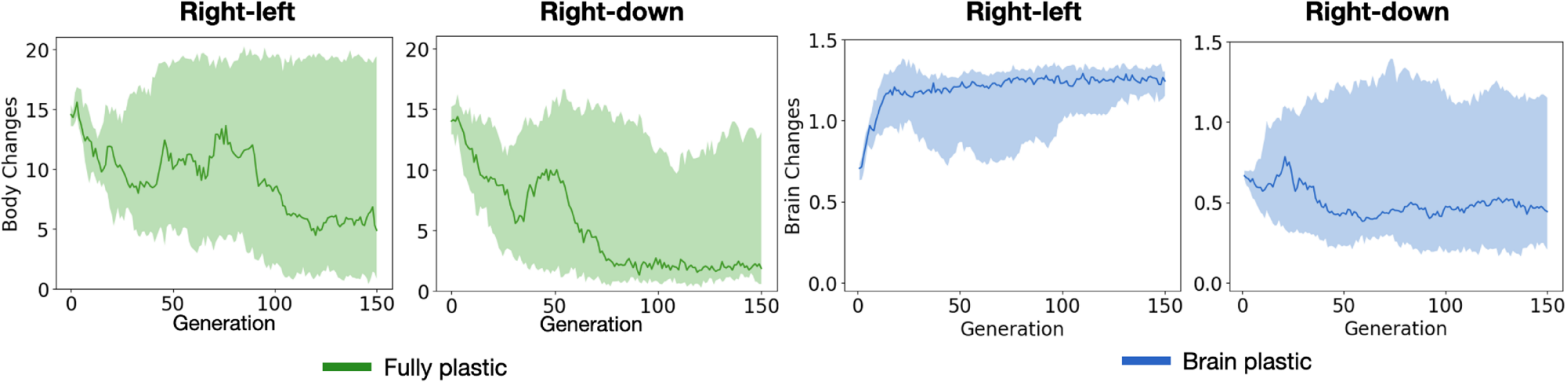
Phenotypic changes. *Body changes* is the tree edit distance, measuring the differences in module configuration between the two possible bodies (one in each condition) of organisms—comparisons were applied only for the *fully plastic* method as it is the only one with body plasticity. *Brain Changes* measures the differences in synaptic connectivity between the two possible brains of organisms—comparisons were applied only for the *brain plastic* method and not for the *fully plastic* method because the latter allows for changes in brain morphology, which would make comparisons difficult. The calculations of the measures are available in Supplementary Information (Phenotypic changes).

**Figure 8:**
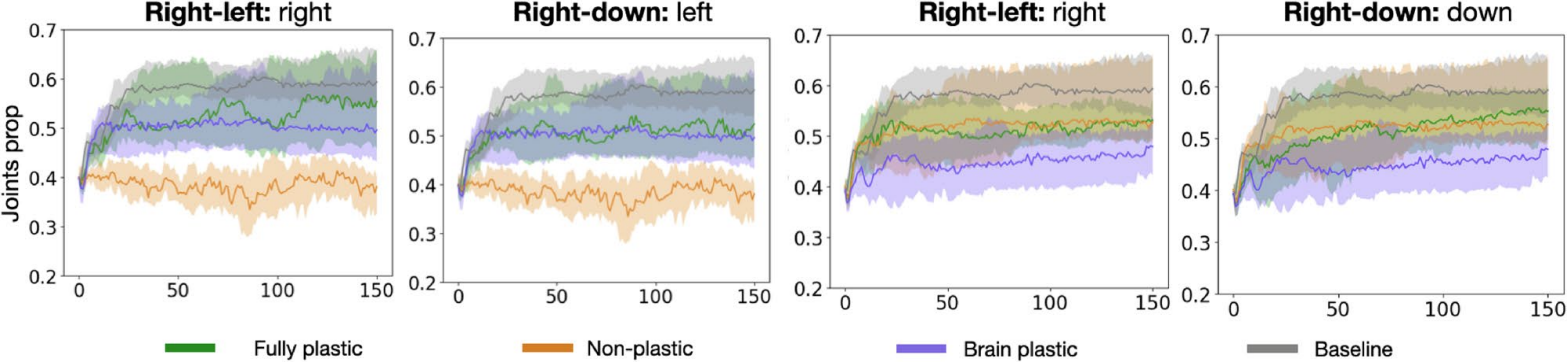
Body trait comparison. *Joints prop* measures the proportion of motor joints relative to the number of modules in the body.

Finally, it should be highlighted that endowing organisms with plasticity in both their bodies and brains did not bring about any significant behavioral differences when compared to endowing organisms with brain plasticity alone. The benefits (or the lack thereof) were the same regardless of the extent to which plasticity was allowed. The idea that plasticity was not necessary in the body is further evidenced by comparing the bodies before and after regulating plasticity—at the moment when the environment changes and the phenotype develops. The curves of body changes in Fig. 7 show that as the evolutionary search progresses, there is no pressure for increasing plastic changes in the body. Moreover, despite high variability, the average number of body changes decreases throughout the search. Predictably, this occurs more intensely in the *right-down* pair, which makes sense because this pair is expected to benefit less from plasticity. Notably, this reduction does not necessarily indicate a selective pressure to reduce body plasticity per se, but rather could derive from pressure for specific body traits that are optimal for locomotion. For example, in both directions of each directional pair, there is pressure for the proportion of joints in the body to increase, and they increase at similar levels for these directions (Fig. 8).

**Figure 9:**
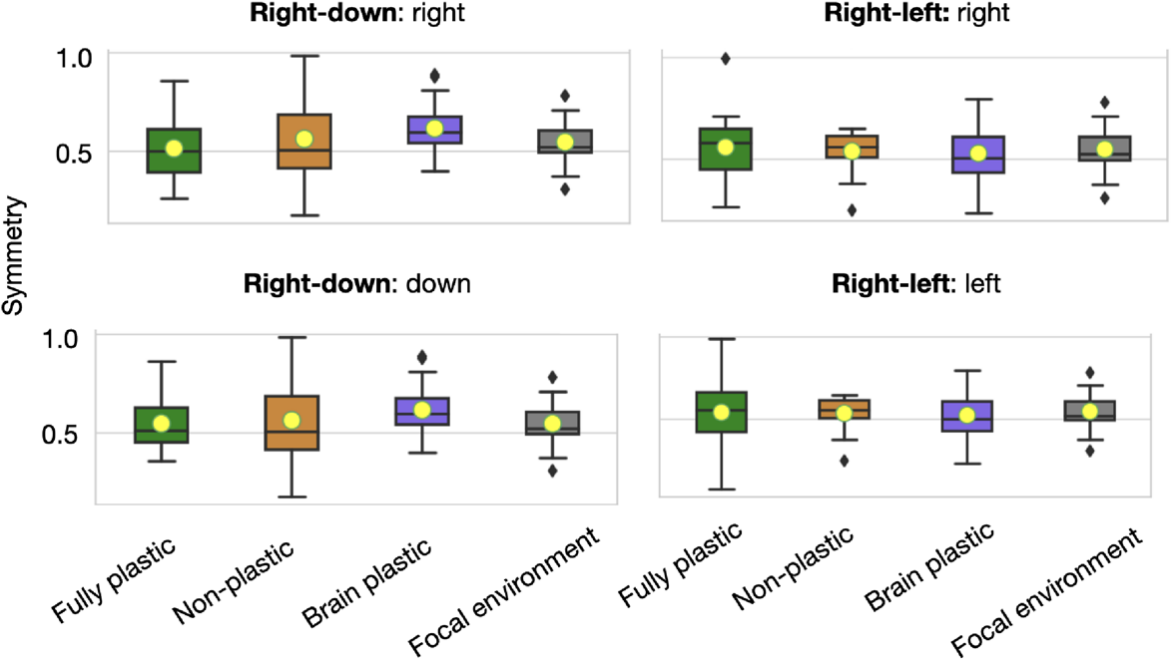
Emergent body traits in the final generation. For all different setups, the symmetry regarding how limbs arrange around the head of an organism is, on average, 50% of the maximum it could be. The charts showing both the progression and distribution of other body traits are available in the Availability of materials and data. The body traits were measured using descriptors proposed in previous research^53^.

**Figure 10:**
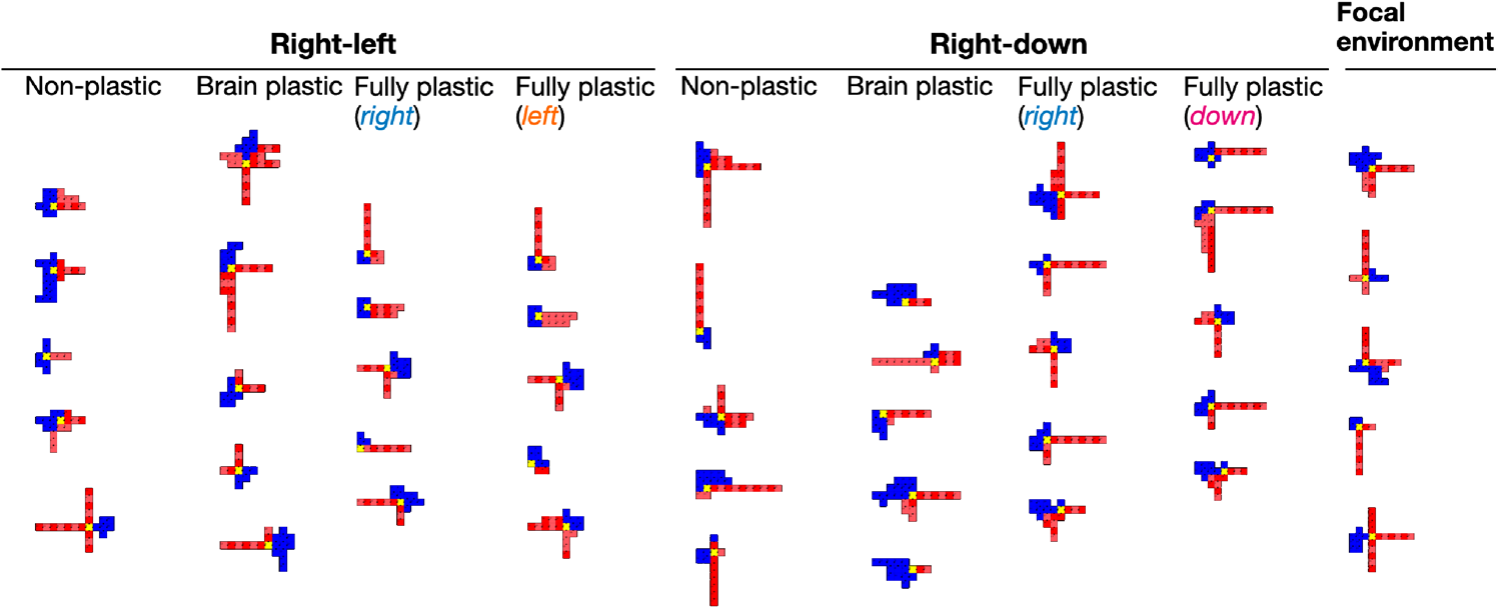
2D top-down illustrations of the body morphologies. Includes the best organism in the final generation of each independent experiment. Visual inspection shows diverse emergent morphologies while there appear to be no obvious differences among them in the different setups. The figure includes five arbitrary experiments, while the illustrations for the other experiments are available in Supplementary Information, Fig. 5.

Moreover, when comparing multiple body traits (body symmetry, number of limbs, proportion of joints, body size, etc.) between the populations produced by the focal environment and those produced by the plastic methods, no convincing evidence was found that their bodies differed (Fig. 9). The same holds when comparing the methods to each other in any direction. Although some traits presented significant differences, after Bonferroni corrections, almost all cases became insignificant. It is important to highlight here that the convergence to these traits is not particularly due to any obvious developmental bias, insofar as the resulting organisms appear very diverse (Fig. 10).

Obtaining comparable traits for both behaviors (directions) is unsurprising given that changing the direction does not truly change the task. Nevertheless, the occurrence of this same effect for all methods should not be taken for granted because the challenge was composed of a pair of directions that required distinct behavior: it was not simply directed locomotion but also included the need for behavioral change during the lifetime of the organism. In this scenario, it is not unreasonable to imagine that when only brain plasticity is possible, certain body traits will be favored in coping with directional changes, e.g., a tail used for propulsion might become inconvenient when the direction changes. Nonetheless, it is interesting that such favoring was not found: brain plasticity itself could control limbs according to the behavioral needs of each direction.

Finally, contrary to the plastic body changes, the occurrences of plastic changes in the brain increased across the evolutionary search for the *right-left* pair (Fig. 7), which is the pair that benefited from brain plasticity. Most of this increase occurred early (around generation 25) and consistently across the experiments, whereas low variability occurred towards the end. This was expected since brain plasticity presented benefits for this pair. Regarding the *right-down* pair, there was no clear pressure to increase brain changes, and again, this makes sense because this pair was expected to benefit less from plasticity.

## Discussion

This study investigated the environmentally regulated phenotypic plasticity of evolvable digital organisms. These organisms had to cope with two different environmental conditions (behavioral needs) during the span of their lifetime. Their traits and behaviors were compared to those of organisms that did not possess phenotypic plasticity. In addition, both these plastic and non-plastic organisms were compared to (non-plastic) organisms that had to cope only with a focal environment.

The results demonstrated a case in which plasticity was beneficial in comparison to non-plasticity and a case in which it did not bring significant improvements. In both of these cases, plastic organisms were unable to achieve fitness comparable to that of the genotypes evolved in the focal environment. Moreover, the body traits of the plastic organisms presented no difference from the traits of organisms that evolved only in the focal environment: plasticity thus did not present *plasticity limits* in the body phenotype. It must be considered, however, that this observation is restricted by the current challenges associated with measuring phenotypic traits. In both biology and artificial life, organisms are composed of diverse phenotypic traits from which a multitude of aspects might be derived. Hence, although this study has used multiple metrics to investigate body traits, they might not necessarily capture every aspect of a particular body morphology. Furthermore, metrics to describe brain structure are still lacking because of the difficulty of formulating meaningful metrics from the control (brain) architecture utilized. Therefore, it is possible that trait differences between the plastic organisms and organisms from the focal environment exist but are not reflected in the current metrics. Such hypothetical differences could include plasticity limits, possibly derived from plasticity costs^17^.

The original hypothesis put forward here was that the potential benefits of plasticity might be undermined by plasticity-related genetic costs. The results support this hypothesis, insofar as plastic organisms can obtain the traits of organisms from the focal environment, but there are deficits in fitness. Note that costs and limits can be closely intertwined^17^ and difficult to measure. Nevertheless, whether it is assumed that plasticity presents costs or limits, plasticity constraints are evidenced by fitness deficits.

These deficits occur in both of the tested scenarios: *right-left*, where the desired behaviors are mutually exclusive, and *right-down*, in which the behaviors are only somewhat contradictory. The two setups are complementary: the results from *right-down* alone could leave one wondering if fitness deficits are not simply a result of having to cope with multiple conditions while the regulatory system is idle. However, the *right-left* pair demonstrates a case in which the lack of plasticity results in much more severe fitness deficits. This clarifies that plasticity is functional but leads to deficits and that depending on the expected gain, there might be no (additional) benefit of plasticity.

Importantly, the only plasticity-related costs possible within the utilized artificial life system were genetic costs, which prevented confounding effects that could be generated by other classes of costs, e.g., production costs. In such a setup, the observed results suggest that these deficits derive from genetic costs. Considering that pleiotropy and epistasis are known in the literature to increase landscape ruggedness^54,55^, this suggestion is further evidenced by the landscape analysis, which demonstrated plasticity increasing ruggedness in the landscape.

At this point, the limitations of these conclusions must be considered carefully. Although genetic costs are the only class of cost allowed in the system, it is possible that the observed deficits are derived from some other emergent mechanism resulting from plasticity, e.g., the presence of plasticity could perhaps affect the genomic organization regardless of pleiotropy and epistasis. Nevertheless, one might go as far as to hypothesise that the gene interactions (epistasis) and gene reuse (pleiotropy) between what codes for regulatory capacities and what codes for traits might explain the ruggedness of the inspected landscapes and therefore the resulting deficits.

For example, a particular gene might both contribute to the ability to produce a specific trait and regulate the expression of another trait. In this scenario, it is possible that a mutation of this gene could increase the quality of a trait while also resulting in the incorrect expression of another trait. If this is true, then it could mean that mutations in genes that code for regulatory capacities cause frequent and severe ‘shifting’ of phenotypic traits that are dependent on this gene. Some examples include mismatch between body and brain traits or the improvement of one body part while another body part is worsened. This hypothetical dynamic might explain why the plastic landscapes are more rugged than the non-plastic landscapes: if important traits share genetic material with crucial regulatory capacities, then there might be highly specific combinations of (good) traits that can be produced without disrupting regulation. Such dynamics are comparable to the well-known challenge in ALife of jointly evolving the body and brain as opposed to evolving only the brain. Although the final populations converge and present morphological traits that are not distinguishable from the traits of populations that evolved in the focal environment, the observed ruggedness suggests that such dynamics could take place before convergence, resulting in fitness deficits. Additionally, mismatches for the final populations might be present in the brain, which lacks metrics for measurement and analysis. One analogous example in biology is how the plasticity of *Arabidopsis thaliana* regarding flowering time leads to negative pleiotropic effects concerning water usage efficiency, which results in maladaptive late-flowering phenotypes^24^.

To conclude, this investigation has shed light on how the genetic costs of phenotypic plasticity might manifest in an ALife system. Although the simplifications of the system make it difficult to evaluate the biological implications of the results, this work contributes to the discussion regarding genetic costs. Crucially, further research is needed to guarantee that the observed effects unequivocally derive from genetic costs and not from some other artifact related to the plasticity mechanisms. Regarding this concern, one useful investigation would be to measure pleiotropy and epistasis directly and analyze how they take place through the evolutionary process in the different setups. These measurements are not feasible within the current ALife setup because the genetic representation utilized is (structurally) an Artificial Neural Network, making gene interactions very difficult to capture and interpret. In future work, it would be interesting to investigate the application of a more biologically plausible and interpretable representation instead, e.g., Gene Regulatory Networks (GRN). This future work would face an extra challenge because analyzing such interactions can be computationally expensive. Additionally, future work should investigate other types of costs in isolation and interaction with each other, which should also address the difference between costs when plasticity is possible in both body and brain and when it is possible only in the brain. This is relevant because rewiring the brain might be less costly than achieving morphological changes^56^.

## Methods

### Genotype, phenotype, and regulation

**Figure 11:**
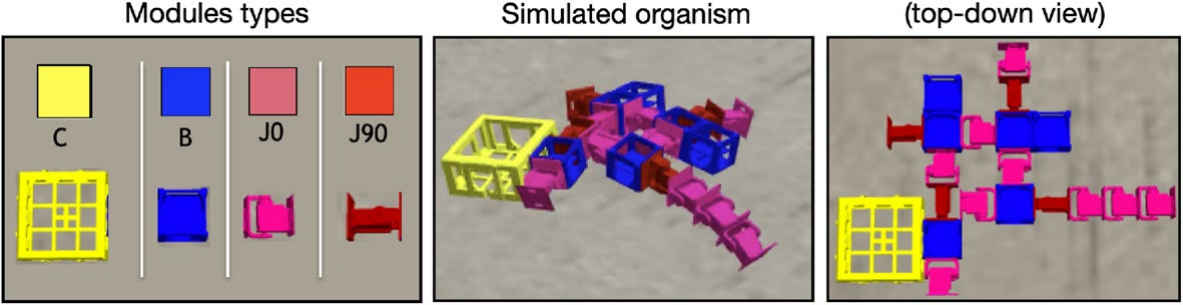
Body morphology. The frame on the left shows the possible modules^59^ with which the body of a robot organism can be composed. C is the core component, which carries the controller electronic board. B is a structural brick. J is a joint with a servo motor—0 and 90 refer to the degrees of rotation of the joint in relation to its parent module. The core component and bricks have four lateral slots for attachment. The joints have only two lateral (and opposite) attachment slots. Any module can be attached to any other module through its slots. The modules allow for attachment on their laterals but not on the top or bottom.

The artificial organisms studied in this work are digital modular robots that possess a body and a brain. The body is composed of a group of assembled modules. The types of modules that can compose a body are shown in Fig. 11, and the body morphology is optimized via evolution. The brain (controller) of an organism is a type of network called a Central Pattern Generator (CPG), and its parameters are also optimized via evolution. “CPGs are neural networks that can produce rhythmic patterned outputs without rhythmic sensory or central input”^57^. In this way, the CPG (brain) produces cyclical sequences of values, and these values are converted into commands that control the motors in the body of the organism. While different types of CPGs exist, the current system uses Ordinary Differential Equations (ODEs), i.e., differential CPGs^58^. Each controller comprises one or multiple differential oscillators, which are pairs of neurons that recurrently connect to each other. In addition, neighboring oscillators also recurrently connect to each other as well.

The CPG architecture of an organism is closely coupled with its body morphology: there is one oscillator for each servo motor. In this way, the controller of an organism consists of one or more differential oscillators, while each of these oscillators comprises a pair of interconnected neurons. For example, the pair of neurons *O*3 and *N*3 forms an oscillator for the motor localized at the point $(x=3,y=2)$ of the grid (see Supplementary Information Fig. 7). These coupled neurons generate oscillatory patterns by calculating their changes of activation at a time step *t* using the following differential equations:

1 $$\begin{aligned} \frac{dx_{(i, t)}}{dt}=w_{y_{i}x_{i}}y_{(i, t-1)} \quad \text {and}\quad \frac{dy_{(i, t)}}{dt}=w_{x_{i}y_{i}}x_{(i, t-1)}, \end{aligned}$$

where *x* and *y* represent neurons *O*3 and *N*3 respectively, and *w* represents the weight of a connection between two neurons; the values of these neurons are updated through the following expressions:

2 $$\begin{aligned} x_{(i, t)} = x_{(i, t-1)} + \frac{dx_{(i, t)}}{dt} \quad \text {and}\quad y_{(i, t)} = y_{(i, t-1)} + \frac{dy_{(i, t)}}{dt}. \end{aligned}$$

In each time step of the organism’s lifetime, the values of the *N* neurons are clipped between $-1$ and 1 and then sent to the respective motors.

The genotypes of the organisms are represented here using Compositional Pattern Producing Networks (CPPNs): one CPPN is utilized for the body and a separate one for the brain. CPPNs^60,61^ are graphs that generate structures and allow the simulation of gene reuse and gene interaction without the need for local interactions and temporal unfolding. A structure is generated through a CPPN by inputting a context to the network (often geometry-oriented) related to the structure, such as, for example, coordinates, and then using the outputs of the network to define the building blocks of this structure. The mapping of the genotype into the phenotype is illustrated and explained in Supplementary Information Figs. 6 and 7 for the body and brain respectively.

Additionally, the *environmental regulation* of *phenotypic plasticity* is enabled by including extra inputs in the CPPN. These inputs act as states of the environment that regulate the expression of genetic material in the phenotype and are referred to as input *S* in the illustrations of Supplementary Information Figs. 6 and 7. The inputs are provided as signals that come from a ‘central system’. The signal has a value of 1 when behavior A is desired and a value of $-1$ when behavior B is desired. This way, when querying the genotype CPPNs to construct the body and brain structures, the values of these additional inputs affect their construction. The addition of these inputs increases the size of the CPPN, and the genes that connect these inputs to the rest of the network evolve in an intertwined manner with other genes. Therefore, connections (genes) involved in regulatory capacities can be reused (pleiotropy) and also interact (epistasis) with genes that code for traits. At the same time, CPPNs have the potential for modularity, which has been argued to favor the evolution of plasticity by reducing pleiotropic constraints^20^.

In summary, the genotype of an individual is composed of three components: the brain genotype (CPPN), the body genotype (CPPN), and a random seed used in the genotype-phenotype mapping of the body. The seed is necessary to guarantee heritability in a context in which there is randomness involved in the mapping. This randomness aims to address or prevent any developmental biases. The seeds are not mutated, though, since mutating a random seed would result in an unacceptably low locality, i.e., small changes in the genotype that could result in enormous changes in the phenotype. Hence, in the initial population, different random seeds are generated and then inherited from then on.

### Evolutionary search and mechanisms

A standard evolutionary computing approach was utilized^34^. In each experiment, a population of virtual organisms evolved for 150 generations. Overlapping generations were utilized with a population size $\mu$ = 100. In each generation, $\lambda$ = 100 offspring were produced via replication and mutation. Individuals were selected for replication by binary tournaments. From the resulting set of $\mu$ parents plus $\lambda$ offspring, 100 individuals were subsequently selected for the next generation using binary tournaments. The fitness function used for each direction measured the speed (cm/s) toward the due direction. The fitness was calculated by evaluating an organism for 30 s. Each organism was evaluated for each one of the desired directions independently, while their fitness values were consolidated using Pareto dominance. In this way, the final fitness function counted the number of dominated individuals using the two fitness values (one from each direction). As one would expect, this consolidation was not necessary for the focal environment experiment.

### Supplementary Information

Supplementary Information.

## Data Availability

The code to reproduce all of the experiments and analysis conducted in this research is available on GitHub https://github.com/karinemiras/revolve2/tree/43c51521bcdc07f31f8f63ca92635f1c5fac5c59/experiments/plasticoding_cppntasks. The data generated in the experiments are available on Kaggle https://www.kaggle.com/datasets/karinemiras/plasticitycosts-miras-23.
