# Supplementary material for: Exploring the costs of phenotypic plasticity for evolvable digital organisms
Source: Sci Rep. 2024 Jan 2;14:108. doi: 10.1038/s41598-023-50683-3 (PMC10761833; doi:10.1038/s41598-023-50683-3)
Supplement: Supplementary file 1 — Supplementary Information. [file 41598_2023_50683_MOESM1_ESM.pdf]

Supplementary Information

Glossary

Key terms used throughout the text are hereby clarified. This is expedient because this work is aimed towards audiences from different backgrounds and because there is sometimes terminology mismatch across different fields.

- **Organism** A virtual artificial creature or robot.
- **Genotype** Artificial DNA of an organism.
- **Phenotype** The body and brain of an organism.
- **Trait** A measurable aspect of the phenotype.
- **Brain** The robot controller.
- **Body** Multiple assembled modules that compose a morphology.
- **Behavior** A measurable phenomenon that emerges from the direct interaction between phenotype and environment.
- **Fitness** A measure of quality calculated *apriori*, as opposed to *aposteri* like in biology. It is used to select organisms for reproduction and survival.
- **Phenotypic plasticity** Environmentally regulated expression of different phenotypes from the same genotype.
- **Acclimatization** Reversible phenotypic changes triggered by environmental cues.
- **Focal environment** A specific environment where organisms evolve.
- **Costs of plasticity** 1) Fitness deficits associated with the plastic genotypes relative to fixed genotypes producing the same mean phenotype in a focal environment. 2) Expressing sub-optimal ('wrong') phenotypes in a given environment.
- **Limits of plasticity** Functional constraints that reduce the benefit of plasticity compared to perfect plasticity.
- **ALife** Involves the design and investigation of artificial living systems in different levels of organisation.
- **Genetic costs** Costs that result directly from (negative) pleiotropic effects of loci affecting plasticity and other traits, or (negative) epistatic interactions among loci affecting plasticity and other loci.
- **Pleiotropy** Genes that are able to influence more than one trait in the phenotype.
- **Epistasis** The interaction between genes so that the effect of a gene might depend on the presence or absence of another gene(s).

Additional figures

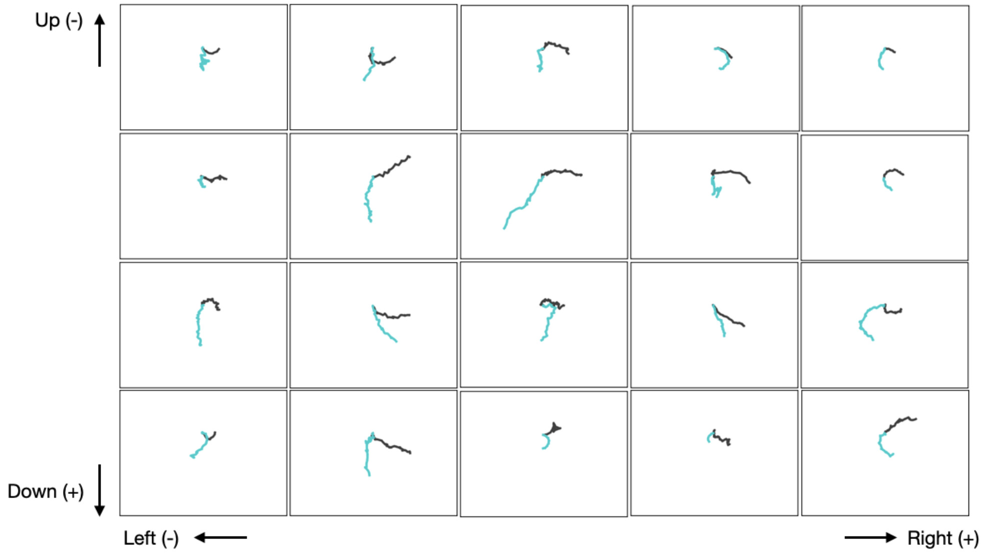

Figure 1. **Trajectories of organisms separated by the independent experiment repetitions for the *right-down* pair when using the *fully plastic* method.**

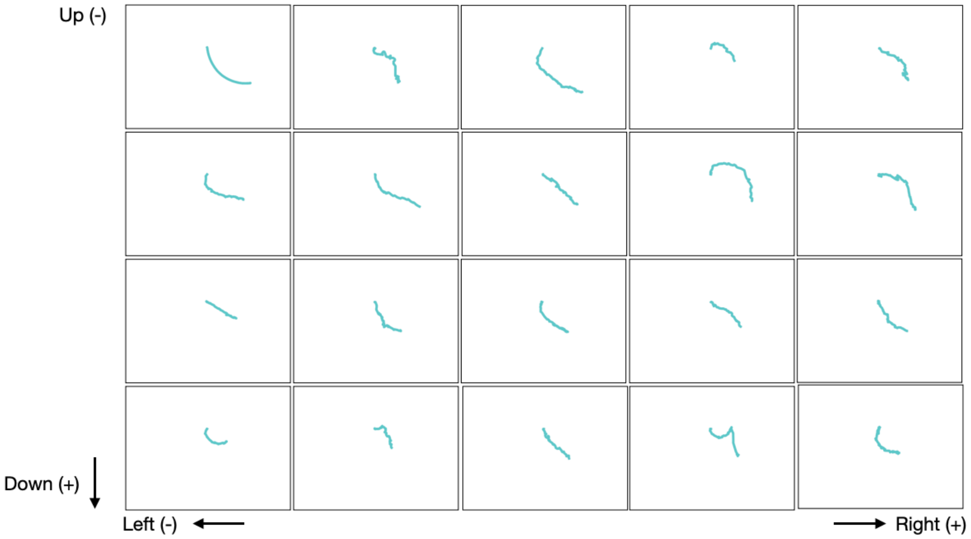

Figure 2. **Trajectories of organisms separated by the independent experiment repetitions for the *right-down* pair when using the *non-plastic* method.**

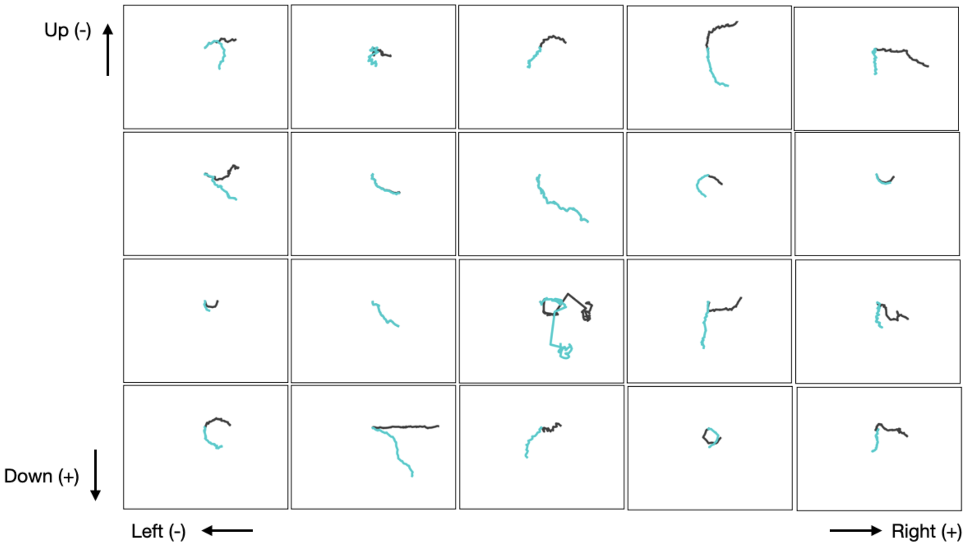

Figure 3. **Trajectories of organisms separated by the independent experiment repetitions for the *right-down* pair when using the *brain plastic* method.**

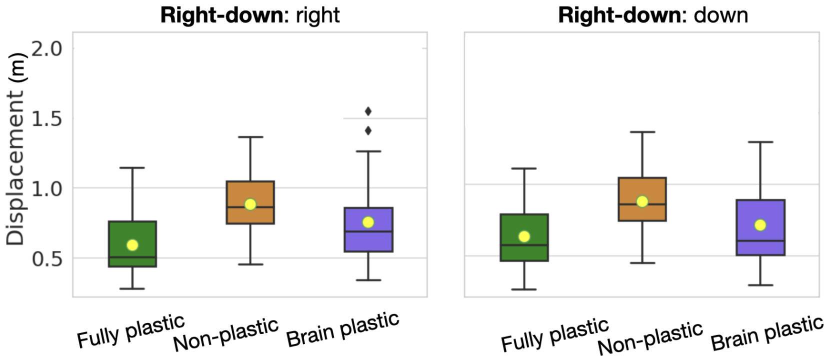

Figure 4. **Average total displacement of the organisms in the final generation.** Calculated as displacement in the x axis plus displacement in the y axis. The non-plastic organisms displace more when summing up x and y displacement.

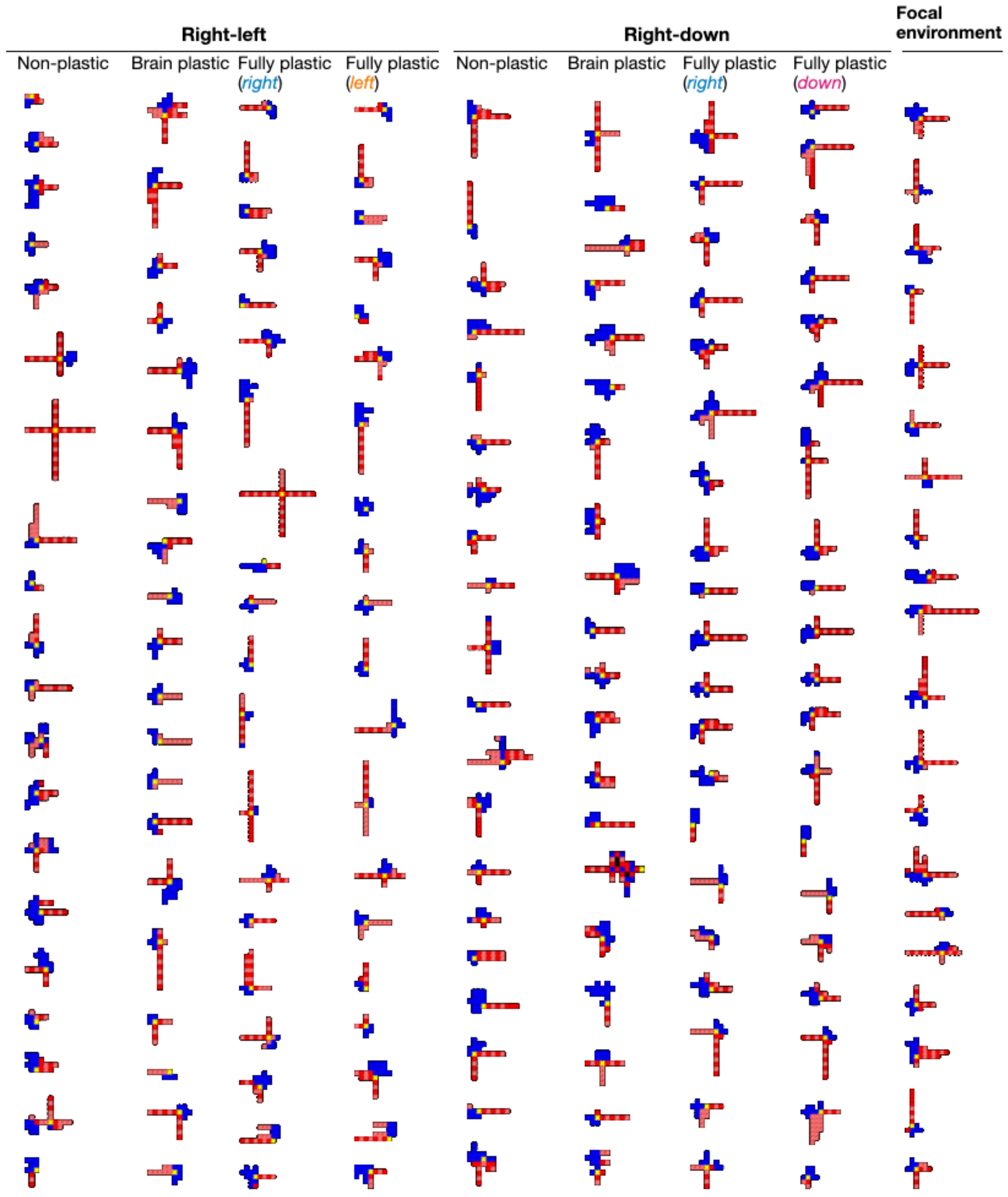

Figure 5. **2D top-down illustration of the body morphology of the best organism in the final generation of each independent experiment.**

Extended methods

Mapping functions

The mapping of the genotype into the phenotype is illustrated and explained in Figs. 6 and 7 for the body and brain respectively.

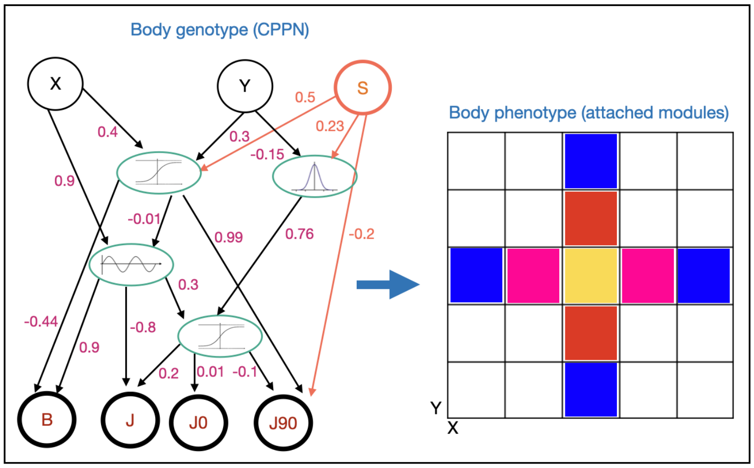

Figure 6. Mapping the body genotype into the body phenotype. A grid with a radius of $r = 15$ points around one central point is defined for the body; the core component is placed in the central point; queries are made to decide which type of module (rotated or not) should be placed in the points ($r = 2$ in the example); querying means providing the x and y coordinates of a point in the grid as input to the body CPPN and then using the output of the CPPN to decide which type of module should be placed in the point - the neurons with the highest value win the decision of using one module or another and also its rotation; a number of $q = 30$ queries is applied; all points that could be occupied using the available attachment slots have an equal chance of being randomly selected to be queried; as new modules are added and attached, new slots become available; if a query tries to place a module in a point already occupied because of a neighbouring slot, or in a point outside of the grid (marginal slots), then this module is not expressed in the body. Input S is a state from the environment used to regulate phenotypic plasticity.

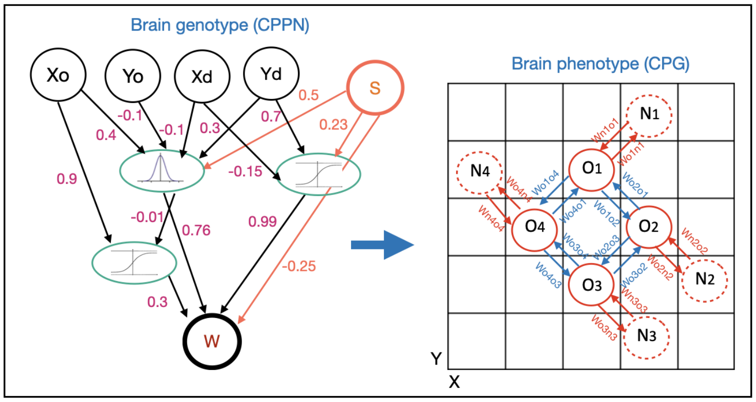

Figure 7. Mapping the brain genotype into the brain phenotype. The brain CPPN is utilized to query the weights of connections between oscillator neurons. The coupled neurons of an oscillator are referred to as (O,N). The querying is carried out in two stages. First: the connection within each oscillator is queried by inputting the coordinates of the module to which the oscillator belongs; this value is then used for the connection from O to N, while its inverse is used for the connection from N to O. Given that the connections are local, both origin and destination coordinates are the same, that is, $(xo = xd, yo = yd)$. Second: the connections between immediate neighbor oscillators are queried; since an oscillator is composed of two coupled neurons (O,N), one of them is chosen as the reference: O (drawn with solid lines); in this instance, the origin and destination coordinates are different, because they connect distinct oscillators. The coupled neurons of differential oscillators are drawn in red, while the connection inter oscillators are indicated in blue. Input S is a state from the environment used to regulate phenotypic plasticity.

Phenotypic changes

The measures of phenotypic changes (from one condition to the other) were calculated by comparing the difference between the phenotype in one environmental condition with the phenotype in another environmental condition.

- *Body Changes* (tree-edit distance): overlay the two bodies using the head as a reference; count disjunction cases - modules that exist in one body but not in the other; for the intersecting modules, count how many are of different types; add up these two numbers.
- *Brain Changes*: overlay the two brains (they have the same neurons and connections); calculate the absolute difference between the weights of each connection; average all the differences.

Procedure for landscape analysis

Using the data generated by the studied methods to analyse the characteristics of the landscapes would be unsuitable because these data are biased towards the difficulty of the landscape itself, that is, rugged landscapes might lead to exploring limited areas of the space when using an objective-oriented search. Therefore, additional experiments were conducted to explore the search space 'agnostically'. This was achieved through Novelty Search (NS)[1], which rather than striving to maximise fitness, instead explores the space to maximise fitness diversity - it discovers solutions that produce fitness values that are maximally different from each other. NS should thus result in the exploration of the space in a non-greedy fashion, which, in turn, allows for a clearer perspective on the landscape.

The fitness function for the NS is defined as $N = n$, where n is a measure of novelty which is calculated as the average distance to the k-nearest neighbours of an organism, for which $k = 10$ and the distance is the Euclidean distance[2] regarding the two desired behaviours: speed in direction A and speed in direction B. The set of neighbors for the comparison is formed by the current population, plus an archive to which 5 organisms of the offspring are randomly added in each generation. The fitness is calculated by evaluating an organism for *30* seconds, precisely as in the other experiments. The evolutionary algorithm utilised is the same as the one described in the previous section, with the exception of the following parameters: population size μ = 30, offspring size λ = 30, and number of generations = 50. The experiments were repeated independently 4 times.

To create the landscape dimensions, the following body trait descriptors were utilised: symmetry, proportion, coverage, length of limbs, number of limbs, branching, joints proportion to body size, bricks proportion to body size, and the ratio between rotated and non-rotated joints. Further details about the trait descriptors are available in the literature[3]. All the descriptions range from 0 to 1. Moreover, all measures were averaged between the two environmental conditions to obtain unified values - this includes the trait descriptors and fitness.

Finally, the (averaged) trait descriptors were reduced to two principal components using Principal Component Analysis. The resulting components PC1 and PC2 were placed on axes x and y of the landscape, respectively, while the (averaged) fitness value was placed on the z-axis.

References

1. Lehman, J. & Stanley, K. O. Abandoning objectives: Evolution through the search for novelty alone. *Evol. computation* **19**, 189–223 (2011).
2. Lehman, J. & Stanley, K. O. Exploiting open-endedness to solve problems through the search for novelty. In *ALIFE*, 329–336 (2008).
3. Miras, K., Haasdijk, E., Glette, K. & Eiben, A. E. Search space analysis of evolvable robot morphologies. In *Applications of Evolutionary Computation - 21st International Conference, EvoApplications 2018*, vol. 10784 of *Lecture Notes in Computer Science*, 703–718 (Springer, 2018).
